# Efficient Scheduling of Heterogeneous Messages in the FlexRay Dynamic Segment

**DOI:** 10.3390/s26103089

**Published:** 2026-05-13

**Authors:** Mingkui Li, Siwen Liu, Haobo Sun, Kaihang Zhang, Yinan Xu

**Affiliations:** College of Engineering, Yanbian University, Yanji 133002, China; mk_li7@ybu.edu.cn (M.L.); 2024050061@ybu.edu.cn (S.L.); 2025010121@ybu.edu.cn (H.S.); 2689332993@ybu.edu.cn (K.Z.)

**Keywords:** intelligent driving technology, FlexRay, dynamic segment, message scheduling, bandwidth utilization

## Abstract

With the rapid development of automotive intelligent driving technologies, the demand for real-time performance and bandwidth in in-vehicle bus networks is increasing day by day. When contrasted with conventional in-vehicle bus protocols like LIN and CAN, FlexRay delivers superior performance in bandwidth capacity, communication latency and data transmission speed. Such prominent strengths establish it as a core technical solution for modern automotive network systems. Targeting the flexible bandwidth characteristics of FlexRay bus systems, this work develops a novel heterogeneous message scheduling algorithm (DHSA) tailored for the dynamic segment of FlexRay. The DHSA enables flexible timeslot and priority configuration for event-triggered and low-priority messages, thereby improving the overall scheduling efficiency of FlexRay bus communication. This work adopts the CANoe.FlexRay simulation tool to construct a dedicated experimental platform and perform comparative simulations for the proposed algorithm. The experimental results show that the bandwidth utilization of the heterogeneous scheduling algorithm proposed in this paper reaches 96.6%, an increase of 13.4% compared to the Earliest Deadline First (EDF) algorithm; meanwhile, the fastest response time of the proposed algorithm is reduced by 50% compared to the EDF algorithm. This study effectively reduces message transmission latency and enhances system real-time performance and determinism, thereby further improving the communication efficiency of the in-vehicle FlexRay bus network.

## 1. Introduction

As automotive electronic systems and data communication technologies continue to evolve rapidly, vehicle control systems are faced with stricter demands for data real-time performance and transmission reliability [1,2]. Traditional LIN and CAN buses have limitations regarding data payload and communication mechanisms. As a low-cost serial communication protocol with limited real-time capability, the LIN bus is widely deployed for automotive body control scenarios, including window lifting and seat position adjustment [3]. The CAN bus is widely used for critical data transmission tasks in vehicle sensor networks and vehicle control systems [4,5]. The FlexRay bus incorporates a dual-channel architecture, where each single channel supports a maximum transmission rate of 10 Mbps, and the combined transmission rate can reach up to 20 Mbps when the two channels operate in parallel mode. Leveraging its inherent strengths in high bandwidth, elevated transmission speed, and robust fault tolerance, this bus protocol is extensively implemented in suspension and chassis control systems [6,7]. As the backbone network for in-vehicle communication, FlexRay plays a crucial role in intelligent connected vehicle fields such as autonomous driving, advanced driver-assistance systems (ADAS), and the Internet of Vehicles (IoV) [8,9].

The FlexRay bus is primarily responsible for data interaction between Electronic Control Units (ECUs). As the number of in-vehicle messages increases daily, the FlexRay bus faces issues such as reduced bandwidth utilization or increased transmission latency when transmitting messages, which affects the stability of in-vehicle systems. To ensure the stable operation of the bus network, FlexRay combines two mechanisms: TDMA (Time Division Multiple Access) and FTDMA (Flexible Time Division Multiple Access). The static segment of FlexRay adopts the TDMA scheduling mechanism. It ensures transmission determinism via preassigned time slots and undertakes the delivery of periodic messages with stringent real-time requirements. The dynamic segment adopts the FTDMA mechanism, supporting priority-based allocation, and is used to transmit non-periodic data and data with significant payload fluctuations [10]. Such a hybrid architecture endows FlexRay networks with both deterministic communication performance and flexible bandwidth management, which lays a solid foundation for efficient message scheduling. Therefore, researching efficient message scheduling algorithms is of great significance for improving the communication performance of the FlexRay bus.

Current studies on FlexRay message scheduling commonly adopt parameter tuning, frame packing and priority adjustment strategies to further boost overall network utilization. Literature proposed a method for sporadic message scheduling [11]. This work leverages the sparse transmission feature of messages in the FlexRay dynamic segment and discretizes this segment into numerous fine-grained mini-slots with short time durations. Whilst maintaining reliable timing characterization, this approach considerably cuts bandwidth overhead and enhances overall computational efficiency. Literature proposed a secure message scheduling scheme for FlexRay in-vehicle networks, optimizing the security, fault tolerance, and real-time performance of message scheduling through methods such as time-slot group authentication, dual-channel transmission, and efficient key updates [12]. Literature proposed a periodic Iterative Learning Control (ILC) message scheduling based on the FlexRay protocol, achieving finite iteration tracking for discrete networks and improving bandwidth utilization and real-time performance by dividing periodic iteration intervals, static/dynamic message transmission segments, and dynamically allocating bandwidth [13]. Literature proposed that a distributed EDF theoretical model based on a global clock is suitable for FlexRay systems [14]. FlexRay possesses a high-precision global clock synchronization mechanism. High-efficiency message scheduling algorithms are effective means to reduce the waste of bandwidth resources.

Efficient transmission of heterogeneous messages has been extensively explored in diverse communication networks. One study presents the PP-RCR cooperative routing algorithm for air–ground integrated ad hoc networks in emergency rescue [15]. By using node position prediction, multi-criteria relay selection, and dynamic copy control, this algorithm realizes efficient and reliable transmission for heterogeneous messages. Another study presents a shortest processing time (SPT) scheduling scheme for heterogeneous SOA-based IoT systems [16]. Using priority queuing and request period optimization, this scheme enhances the transmission stability and service quality of heterogeneous messages.

However, existing FlexRay message scheduling research lacks methods based on FlexRay communication channels, resulting in the failure to fully utilize the two communication channels of FlexRay. Most relevant studies only focus on single-channel priority optimization, deadline-oriented sorting, or frame encapsulation strategies. They ignore the inherent architectural feature of independent transmission in FlexRay dual channels and fail to tap into the potential of dual-channel parallel communication. Meanwhile, traditional FlexRay Identifier (FID) allocation schemes rely on a single metric (e.g., deadline or traffic load), leading to a trade-off dilemma between bandwidth utilization and message real-time performance.

The core technical innovation of this paper is the integration of a dual-channel heterogeneous allocation mechanism and a dual-strategy FID optimization scheme. Specifically, we improve channel utilization by selecting the optimal allocation scheme based on the dual-channel earliest completion time criterion. Furthermore, we propose an adaptive FID allocation strategy that makes joint decisions by considering three critical indicators: deadline constraint, remaining execution time, and traffic load intensity. This integrated method effectively resolves the problems of insufficient channel resource utilization and irrational priority allocation in conventional approaches. It achieves a fundamental improvement in dynamic segment scheduling efficiency from both architectural and strategic perspectives, thereby forming a clear technical boundary with existing single-channel and single-metric scheduling methods.

This study proposes a Heterogeneous Message Scheduling Algorithm for the FlexRay dynamic segment. By employing a heterogeneous allocation method for dynamic segment message communication channels, combined with a Frame ID (FID) allocation module and a dynamic channel allocation module, the proposed algorithm achieves highly efficient message scheduling for the FlexRay dynamic segment. Section 2 introduces the FlexRay bus network. Section 3 proposes the Heterogeneous Message Scheduling Algorithm (DHSA) for the FlexRay dynamic segment. Section 4 evaluates the performance of the heterogeneous message scheduling algorithm. Finally, Section 5 summarizes the research content and draws conclusions.

## 2. FlexRay Bus Network

**Figure 1:**
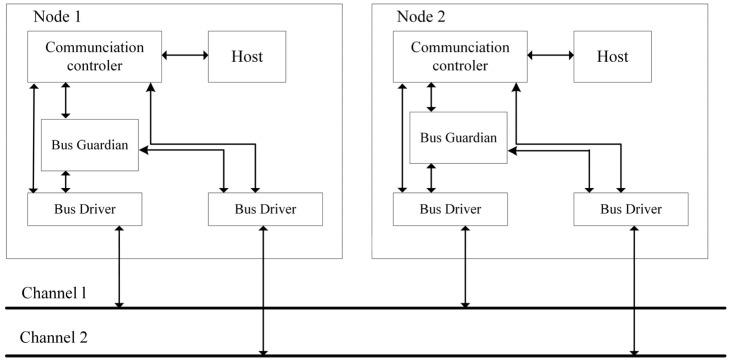
FlexRay Node Structure Diagram.

The FlexRay bus possesses two channels; each channel has a maximum data rate of 10 Mbps, and the total data rate can reach 20 Mbps. The FlexRay node structure includes four main components: Host (Central Processing Unit), Communication Controller, Bus Guardian, and Bus Driver. Figure 1 illustrates the FlexRay node structure. Node 1 and Node 2 are connected to Channel 1 and Channel 2 via their respective Bus Drivers and Bus Guardians [17].

**Figure 2:**
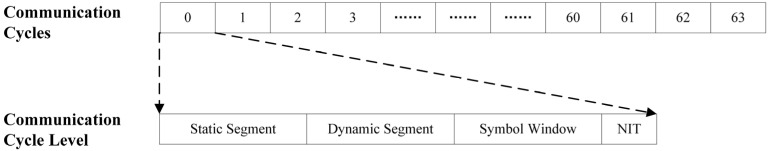
FlexRay Communication Cycle.

A FlexRay communication cycle consists of four components: the Static Segment, Dynamic Segment, Symbol Window, and Network Idle Time (NIT), as shown in Figure 2. The static segment uses a time-triggered mode, strictly allocating fixed transmission slots [18,19]. The dynamic segment supports event-triggered communication. Relying on mini-slots, it enables flexible resource adjustment in response to real-time communication demands. The symbol window is used to transmit synchronization and management information. The network idle time is a brief period used to ensure that no nodes in the network are transmitting data. These four parts constitute the complete structure of a FlexRay communication cycle, ensuring the orderly conduct of the entire network communication.

### 2.1. FlexRay Data Frame Structure

**Figure 3:**
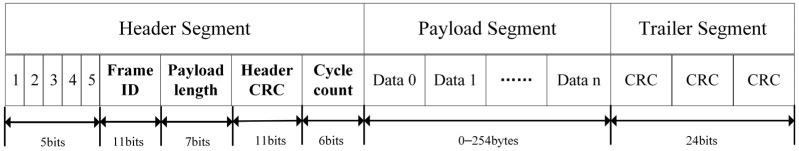
FlexRay Frame Structure.

The FlexRay data frame is composed of three parts: the Header Segment, Payload Segment, and Trailer Segment [20]. As shown in Figure 3. The first 5 bits of the Header Segment are, in order: the reserved bit, payload preamble indicator, null frame indicator, sync frame indicator, and startup frame indicator; the FID occupies 11 bits; and the payload length in the frame header occupies 7 bits. The Frame ID (FID) is used to identify the position of the data frame within the communication cycle. The Payload Segment is used to represent the number of bytes of valid data in the payload section, ranging from 0 to 254 bytes. The Header CRC is 11 bits long and contains a Cyclic Redundancy Check. The transmitting node calculates the CRC value when sending a data frame, and the receiving node recalculates it after receiving the frame and compares it with the received CRC value to judge whether the received data frame is correct. In the dynamic segment, the Payload Segment length can be configured; the first two bytes serve as the message ID to define the content of the data segment. The Trailer Segment is a 3-byte CRC checksum.

### 2.2. Transmission Characteristics of the FlexRay Dynamic Segment

The FlexRay dynamic segment operates based on the Flexible Time Division Multiple Access (FTDMA) scheme. Message transmission is realized through mini-slots with a uniform and fixed time length. The FlexRay dynamic segment consists of multiple dynamic slots, with the mini-slot serving as its fundamental constituent unit. Each mini-slot is constructed with Macroticks (MT), while the time length of a dynamic slot adjusts dynamically according to the size of transmitted messages. In the dynamic segment of the FlexRay bus, message transmission relies on dynamic frames.

**Figure 4:**
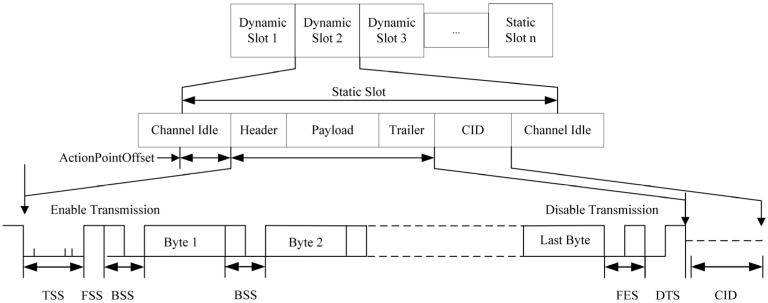
FlexRay Dynamic Frame Encoding Rules.

FlexRay data frames are divided into static frames and dynamic frames [21]. Dynamic frames are the basic units for message transmission in the FlexRay bus dynamic segment. The encoding rules for FlexRay dynamic frames are shown in Figure 4. During encoding, the Transmission Start Sequence (TSS), Frame Start Sequence (FSS), Byte Start Sequence (BSS), and Frame End Sequence (FES) must be added; additionally, a Dynamic Trailing Sequence (DTS) is added to message frames in the dynamic segment.

Let the message set of the dynamic segment be $M_{DS}=\left\{m_{1},m_{2},\ldots ,m_{n}\right\}$, where $m_{j}$ is the *j*-th dynamic message, there are n dynamic messages in total, and $m_{j}$ in $M_{DS}$ When the length of message $m_{j}$ is $w_{j}$ bits, the length of the encoded dynamic frame is represented as $L\left(m_{j}\right)$, as shown in Equation (1).

(1) $$L(m_{j})=TSS+FSS+80bits+1.25w_{j}+FES+DTS$$

Among them, the 80-bit value accounts for the total length of fixed-size fields within both the header and trailer segments of a FlexRay dynamic frame. These fields encompass reserved bits, the frame identifier (FID), payload length indicator, and header cyclic redundancy check (CRC) in the frame header section, as well as the fixed checksum bits in the frame trailer. Defined explicitly as a fixed-length overhead by the FlexRay protocol specification, this 80-bit segment is entirely independent of the actual payload length of the transmitted message.

The FlexRay dynamic slot length $L_{DS\left(m_{j}\right)}$ is shown in Equation (2).

(2) $$L_{DS\left(m_{j}\right)}=1+ceil\left(\left(\left(L\left(m_{j}\right)+1\right)\times t_{bit}\right){\left(t_{MT}\times \left(1-CDM\right)\right)}^{-1}\right)+DSIP$$

Among them, $L_{DS\left(m_{j}\right)}$ denotes the number of minislots occupied for transmitting a dynamic message. Herein, ceil refers to the ceiling function, $t_{bit}$ represents the transmission time per bit, $t_{MT}$ denotes the duration of a single Macrotick, and $t_{MS}$ refers to the duration of a single minislot. CDM, short for Clock Deviation Margin, is used to compensate for clock synchronization offsets between nodes, with typical values ranging from 0 to 1. DSIP, the abbreviation for Dynamic Slot Idle Phase, denotes the protocol-mandated idle interval, which typically takes a value of 0, 1, or 2 minislots in FlexRay dynamic segment communication.

The length of the FlexRay dynamic segment is shown in Equation (3), where $N_{MS}$ is the number of mini-slots contained in the dynamic segment.

(3) $$L_{DS}=N_{MS}\times t_{MS}$$

The FlexRay network utilization is shown in Equation (4). Here, N is the number of communication cycles required to transmit all dynamic messages, and $U_{DS}$ refers to the percentage of the total bandwidth occupied by the bandwidth required for message transmission.

(4) $$U_{DS}=\sum_{j=1}^{n}L_{DS}\left(m_{j}\right)∕L_{DS}\times N$$

Bandwidth utilization of the FlexRay dynamic segment serves as a core metric for evaluating the overall operational efficiency and bus performance. Higher bandwidth utilization contributes to better operational performance of the entire FlexRay communication system. The bandwidth utilization is closely related to the dynamic segment length. An excessively short dynamic segment will raise message latency and thereby degrade the real-time transmission capability. Since there is uncertainty in the sending moment of messages in the dynamic segment, it is necessary to analyze the Worst-Case Response Time (WCRT) of each message.

### 2.3. Worst-Case Response Time of the FlexRay Dynamic Segment

The Worst-Case Response Time (WCRT) of the FlexRay dynamic segment acts as a critical metric to evaluate the bus’s real-time communication performance. It characterizes the maximum time consumption for dynamic messages from initial transmission request to final delivery under the worst scheduling conditions.

**Figure 5:**
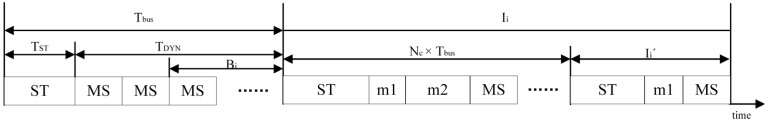
Dynamic Segment Message Response Time.

In practical message transmission scenarios, the magnitude of the worst-case response time directly determines the real-time performance of data messages. If the WCRT of a critical message exceeds the bus limits, the message cannot be transmitted on time, thereby affecting the stable operation of the vehicle. In a high-load communication environment, mini-slots in the dynamic segment are easily occupied by high-priority tasks, leading to increased waiting times for low-priority messages [22]. The dynamic segment message response time is shown in Figure 5.

The worst-case response time of the dynamic segment in the FlexRay bus is expressed by Equation (5). Herein, ${\;T}_{i\;}$ denotes the transmission time of message $m_{i}$, $I_{i}$ represents the blocking delay induced by the static segment and transmissions of higher-priority messages, and $B_{i}$ refers to the bus cycle delay incurred when message $m_{i}$ misses its target transmission minislot.

(5) $${WCRT}_{D}\left(m_{i}\right)=B_{i}+I_{i}+T_{i}$$

The bus cycle delay is shown in Equation (6). Here, $T_{bus\;}$ denotes the total duration of a communication cycle, $T_{ST}$ represents the length of the static segment, $T_{MS}$ signifies the duration of a single minislot, and ${ID}_{m}$ refers to the Frame Identifier (FID) corresponding to a given message. Within the FlexRay dynamic segment mechanism, the core function of the FID is to specify the initial offset position where a message commences minislot contention, rather than to dictate the final transmission sequence of messages. As such, even if message transmission within the dynamic segment does not strictly adhere to the FID sequence, $\left({ID}_{m}-1\right)$ can still precisely quantify the number of minislot offsets from the start of the dynamic segment to the position where the message is permitted to initiate contention.

The expression $\left(T_{ST}+\left({ID}_{m}-1\right)\right)\times T_{MS}$ denotes the total time elapsed from the start of a communication cycle to the position where the message becomes eligible for minislot contention. By subtracting this time duration from the total communication cycle length, the bus cycle delay Bi—incurred when a message misses its intended transmission slot—can be derived. Rooted in the standard rules governing the correlation between FID and minislot offset in the FlexRay protocol, this formula bears a clear physical interpretation and is fully aligned with the formal FlexRay communication protocol specifications.

(6) $$B_{i}=T_{bus}-\left(T_{ST}+\left({ID}_{m}-1\right)\right)\times T_{MS}$$

$I_{i}$ is the delay caused by the static segment and the transmission of higher-priority messages, as shown in Equation (7). Here, $N_{c}$ is the number of cycles where the message cannot be transmitted due to high-priority message transmission, and $I_{i}^{\prime }$ is the time the message is delayed within the transmission communication cycle.

(7) $$I_{i}=N_{c}\times T_{bus}+I_{i}^{\prime }$$

The worst-case response time of the FlexRay dynamic segment indicates the maximum transmission delay encountered during bus communication operations. By analyzing the message transmission time, the delay caused by missed mini-slots, and the blocking time caused by high-priority message transmission, the worst-case response time of the message is calculated. Accurate assessment of the WCRT helps in assigning message priorities, thereby further improving the transmission efficiency of the FlexRay bus.

## 3. Heterogeneous Message Scheduling Algorithm

This paper first investigates the transmission features and worst-case response time of the FlexRay dynamic segment. Combined with the FID assignment and channel allocation modules for dynamic segments, a novel Dynamic Heterogeneous Message Scheduling Algorithm (DHSA) is further proposed. This algorithm addresses the bandwidth utilization of the FlexRay bus while reducing message transmission time.

### 3.1. Architecture of the DHSA

**Figure 6:**
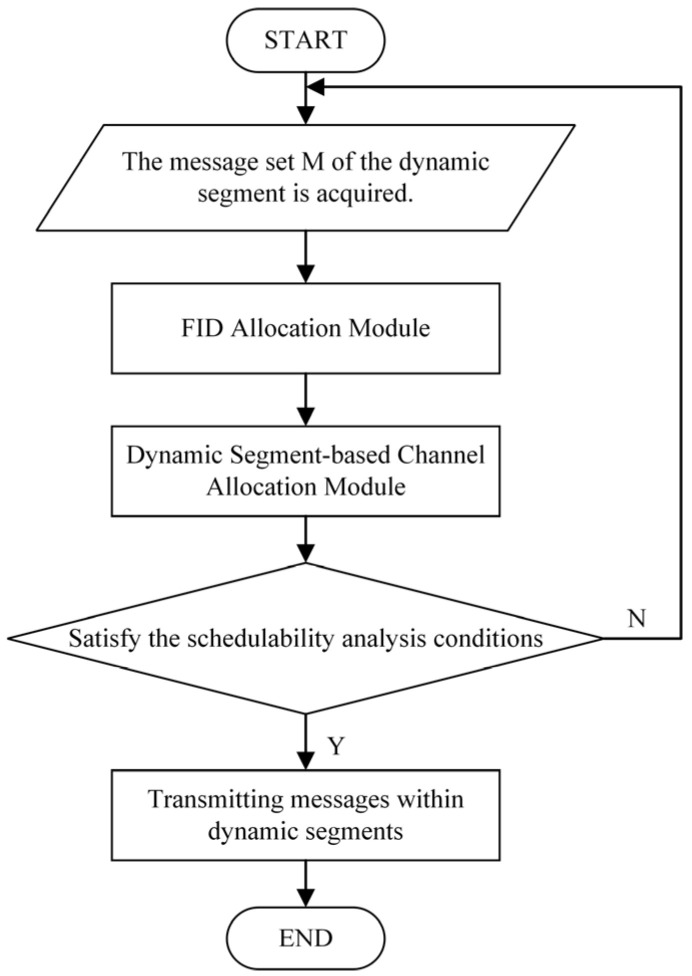
Flowchart of the DHSA.

The DHSA consists of heterogeneous allocation of communication channels, an FID allocation module, and schedulability analysis; its workflow is shown in Figure 6. First, the dynamic segment message set M is obtained from the FlexRay transmitting nodes, and FID allocation is performed according to deadlines, payloads, and remaining execution times. Next, through the dynamic segment channel allocation module, the algorithm traverses the earliest finish transmission times of the dynamic segment messages and selects the channel with the smaller transmission time as the communication channel for message transmission. Finally, the scheduling results are evaluated for schedulability. If the schedulability conditions are met, the dynamic segment messages are transmitted; if the schedulability conditions are not met, the dynamic segment message set is reacquired, and channel allocation is performed again.

### 3.2. Design of the FID Allocation Module

Within the FlexRay dynamic segment, message priority dominates the scheduling sequence of data transmission. The smaller the data FID value, the higher the priority. Therefore, the allocation of message FIDs directly affects the scheduling efficiency of the bus, and an efficient priority allocation method can enhance the bandwidth utilization of the bus.

Based on factors such as message payload length, deadline, and remaining execution time, this study proposes an FID allocation module. As shown in Algorithm 1. First, the dynamic segment message set $M_{D}$ is obtained from the FlexRay transmitting nodes. Second, FID allocation is performed based on the principle of increasing deadlines and decreasing payloads to obtain bandwidth utilization U (line 1); simultaneously, FID re-allocation is performed based on the principle of increasing remaining execution time and decreasing payloads to obtain bandwidth utilization $U^{\prime }$ (line 2). Finally, by comparing the bandwidth utilization of different FID allocation methods, the optimal FID allocation result is obtained.
**Algorithm 1:** FID Allocation Algorithm**Input**: Dynamic segment message set
$M_{D}$**Output:** FID allocation results1: Initialize FIDs based on increasing deadlines and decreasing payloads, and obtain U;2: Reassign FIDs based on increasing remaining execution time and decreasing payload, and obtain $U^{\prime }$;3: **if** $U>U^{\prime }$ **then**4:  Output the initial FID allocation results;5: **else**6:  Output the reassigned FID allocation results;7: **end if**8: **Return** FID allocation results

In Algorithm 1, the dynamic segment message set is $M_{D}=\{m_{1},m_{2},\ldots ,m_{n}\}$, and $d_{m_{i}}$ is the deadline of message $m_{i}$; message priorities are sorted according to the principle of increasing deadlines. When the deadline $d_{m_{i}}$ is less than or equal to $d_{m_{j}}$, the value of *i* is less than *j*, as shown in Equation (8).

(8) $$D_{mi}\leq d_{mj}?j>i:j<I\left(\forall m_{I,j}\in M,i\neq j\right)$$

Data FIDs are reassigned according to the principle of increasing remaining execution time of dynamic segment messages, as shown in Equation (9). Here, ${STT}_{m_{i}}$ is the remaining execution time of dynamic segment message $m_{i}$ When the remaining execution time ${STT}_{m_{i}}$ is less than or equal to ${STT}_{m_{j}}$, the value of i is less than j.

(9) $${STT}_{mi}\leq {STT}_{mj}?j>i:j<i\left(\forall m_{i,j}\in M,i\neq j\right)$$

${STT}_{m_{i}}$ is calculated from the message deadline $d_{m_{i}}$ and the worst-case response time ${\;WCRT}_{m_{i}}$, as shown in Equation (10).

(10) $${STT}_{mi}=d_{mi}-{WCRT}_{mi}$$

The message set sorted by deadline or remaining execution time is defined as ${M_{D}}^{\prime }$. Next, the messages in set ${M_{D}}^{\prime }$ are sorted according to the principle of decreasing payload. $L_{m_{i}}$ is the payload of message $m_{i}$, as shown in Equation (11).

(11) $$L_{mi}\geq L_{mj}?j>i:j<i\left(\forall m_{i,j}\in M,i\neq j\right)$$

By rationally assigning priority levels to all messages transmitted in the dynamic segment, the FID assignment module can substantially improve the bandwidth efficiency of the FlexRay bus. This FID assignment module evaluates the bandwidth performance of various allocation strategies and selects the most suitable FID configuration as the final solution. This mechanism guarantees the preferential transmission of high-priority messages, which reduces the worst-case response time and further enhances the overall communication performance of the dynamic segment.

### 3.3. Heterogeneous Allocation of Communication Channels

The FlexRay bus architecture contains two independent communication links; this paper designs a channel heterogeneous allocation algorithm specifically for the dynamic segment, which can maximize the utilization efficiency of the dual channels, as shown in Algorithm 2. First, using the output of the FID allocation algorithm as input, the communication duration $T_{i}$ required for each message is calculated (line 1). Second, the algorithm estimates the earliest transmission finish time for each message $m_{i}$ on channels CH1 and CH2, denoted as $EFT\left\{m_{i},{CH}_{1,2}\right\}$. The specific allocation criterion is: if the earliest finish time of message $m_{i}$ on channel 1 is earlier than on channel 2, it is allocated to channel 1 for transmission. Conversely, it is allocated to channel 2 (lines 3–10). Finally, the determined channel allocation scheme is output. See Algorithm 2 for the detailed process.
**Algorithm 2:** Dynamic Segment Heterogeneous Channel Allocation Algorithm**Input:** FID allocation results**Output:** Channel allocation results1: $T_{i}=\frac{L_{m_{i}}}{R}$;2: **for**
$m_{i}\in$ FID allocation results **do**3: EST(mI,CH1,2)=max{ETA[CH1,2],EFT(mj,CH1,2)+Timj∈pred(mi)max};4: $EFT\left(m_{i},{CH}_{1,2}\right)=w_{i,{CH}_{1,2}}+EST\left(m_{i},{CH}_{1,2}\right)$;5:    **if** $EFT\left(m_{i},{CH}_{1}\right)<EFT\left(m_{i},{CH}_{2}\right)$ **then**6:     Assign $m_{i}$ to Channel 1;7:    **else**8:     Assign $m_{i}$ to Channel 2; 9:    **end if** 10: **end for** 11: **Return** Channel allocation results

$T_{i}$ is the communication time of message $m_{i}$, as shown in Equation (12). Where $L_{m_{i}}$ is the payload size of message $m_{i}$, and R is the transmission rate of the FlexRay bus.

(12) $$T_{i}=L_{mi}∕B$$

$EFT\left(m_{i},{CH}_{1,2}\right)$ is the earliest finish transmission time of message $m_{i}$ on channel ${CH}_{1,2}$, as shown in Equation (13). $EST\left(m_{i},{CH}_{1,2}\right)$ is the earliest start transmission time of message $m_{i}$ on channel ${CH}_{1,2}$, as shown in Equation (14). In Formula (14), $pred\left(m_{i}\right)$ denotes the set of messages that have a higher priority than mi. This priority relationship is globally established and permanently fixed upon the completion of FID allocation in Algorithm 1, as opposed to being dynamically computed during the channel assignment phase. Given that a smaller FID value corresponds to a higher message priority, the priority sequence of all messages is fully determined before Algorithm 2 is initiated in the dual-channel scheduling framework. $ETA\left[{CH}_{1,2}\right]$ is the earliest allowable message transmission time on channel ${CH}_{1,2},$ as shown in Equation (15).

(13) $$EFT\left(m_{i},{CH}_{1,2}\right)=w_{{i,CH}_{1,2}}+EST\left(m_{i},{CH}_{1,2}\right)$$

(14) EST(mi,CH1,2)=max{ETA[CH1,2],[EFT(mj,CH1,2)+Tj]mj∈pred(mi)max}

(15) $$ETA\left[{CH}_{1,2}\right]=N_{{CH}_{1,2}}\times T_{bus}$$

where ${CH}_{1,2}$ represents the two communication channels ${CH}_{1}$ and ${CH}_{2}$ of the FlexRay bus. $pred(m_{i})$ represents messages with higher priority than message $m_{i}$. $w_{i,{CH}_{1,2}}$ represents the time for channel ${CH}_{1,2}$ to receive and process message $m_{i}$. $N_{{CH}_{1,2}}$ represents the number of cycles where transmission is impossible on channel ${CH}_{1,2}$ due to high-priority message transmission. $T_{bus}$ represents the time length of the FlexRay bus communication cycle. To determine the timing characteristics of message transmission, this section elucidates the calculation process for the Earliest Start Time (EST) and Earliest Finish Time (EFT) of messages. First, this study calculates the earliest allowable message transmission time $ETA\left[{CH}_{1,2}\right]$ on channel ${CH}_{1,2}$. Second, by comparing $ETA\left[{CH}_{1,2}\right]$ with the earliest finish transmission time of message $pred(m_{i})$, the earliest start transmission time $EST\left(m_{i},{CH}_{1,2}\right)$ of message $m_{i}$ on channel ${CH}_{1,2}$ is obtained. Finally, by adding $EST(m_{i},{CH}_{1,2})$ and the processing time $w_{i,{CH}_{1,2}}$, the earliest finish transmission time $EFT\left(m_{i},{CH}_{1,2}\right)$ of $m_{i}$ on channel ${CH}_{1,2}$ is obtained.

The channel allocation module for the dynamic segment determines the proper transmission channel by comparing the earliest finish time of message delivery across the two available communication channels. When transmitting message $m_{i}$, it compares the earliest finish transmission times $EFT\left(m_{I},{CH}_{1}\right)$ and $EFT\left(m_{I},{CH}_{2}\right)$ on ${CH}_{1}$ and ${CH}_{2}$ and selects the communication channel with the smaller finish transmission time to transmit message $m_{i}$, thereby reducing the message transmission time.

### 3.4. Schedulability Analysis of Dynamic Segment Messages

In the FlexRay dynamic segment, message transmission relies on schedulability analysis. The dynamic segment adopts an event-triggered mechanism, where nodes only compete for the bus when a mini-slot arrives. Accordingly, the transmission feasibility of each dynamic-segment message must be fully evaluated prior to actual transmission. Schedulability analysis is capable of verifying whether individual messages can be delivered within the expected time range, which lays a solid foundation for efficient dynamic segment scheduling.

In the message scheduling process, high-priority messages seize bus resources with priority. This occupation will block the transmission of low-priority data and further extend their queuing delay. Therefore, the schedulability analysis for dynamic segment messages must simultaneously satisfy the following two constraints: First, it must ensure that the message’s worst-case response time does not exceed its deadline, as shown in Equation (16).

(16) $${WCRT}_{D}\left(m_{i}\right)\leq d_{m_{i}}$$

Second, it must guarantee that the earliest start transmission time of the message on the channel does not exceed the latest time point allowed for the node to send messages in the dynamic segment, as shown in Equation (17).

(17) $$EST\left(m_{i},{CH}_{1,2}\right)\leq pLatestTx\left(m_{i}\right)$$

where ${WCRT}_{D}(m_{i})$ is the worst-case response time of dynamic segment message $m_{i}$, $d_{m_{i}}$ is the deadline of message $m_{i}$, $EST\left(m_{i},{CH}_{1,2}\right)$ is the earliest start transmission time of message $m_{i}$ on channel ${CH}_{1,2}$, and $pLatestTx(m_{i})$ is a FlexRay bus parameter representing the latest time point for a node to send a message in the dynamic segment. For every message $m_{i}$ in the FlexRay dynamic segment, to ensure message transmission completion, it is mandatory that the worst-case response time ${WCRT}_{D}(m_{i})$ is less than or equal to the message deadline $d_{m_{i}}$, and simultaneously, the earliest start transmission time of message $m_{i}$ on channel ${CH}_{1,2}$ must be less than or equal to the latest time point for the node to send messages in the dynamic segment.

In terms of schedulability, this paper imposes two core constraints: the worst-case response time of each message must not exceed its assigned deadline, and the earliest activation time of a message on the communication channel must not surpass the maximum allowable transmission moment of the dynamic segment. Together, these dual constraints guarantee that all messages can meet stringent timing requirements and accomplish reliable transmission even under high-load operating conditions.

In terms of computational overhead, the algorithm proposed herein comprises merely two primary steps: message priority assignment and dual-channel selection. Both procedures are executed through the comparison and traversal of intrinsic message attributes, obviating the need for intricate iterative operations or large-scale numerical computations. Boasting low overall computational complexity and high execution efficiency, the algorithm is well-suited to meet the strict demands for real-time performance and lightweight operation inherent to in-vehicle embedded systems.

## 4. Simulation Experiment

CANoe is a powerful tool designed, developed, and used for simulation and testing of bus networks by Vector Informatik GmbH, Stuttgart, Germany (https://www.vector.com). CANoe supports various bus protocols such as LIN, CAN, FlexRay, MOST, and Ethernet. CANoe.FlexRay is an extension module of the CANoe platform, specifically used for the development and simulation of the FlexRay communication protocol.

**Figure 7:**
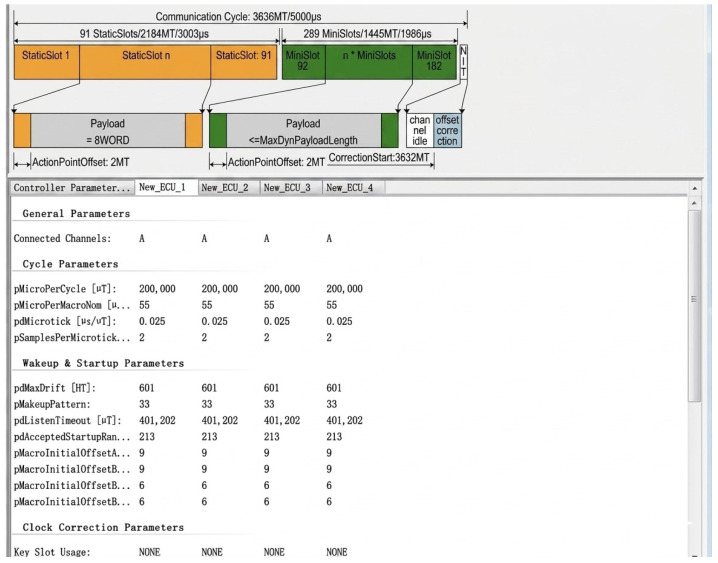
Network Designer.FlexRay Interface.

In this study, the Network Designer.FlexRay tool is employed to construct and manage the communication scheduling tables. As illustrated in Figure 7, this configuration explicitly defines the partitioning of static slots and dynamic minislots within a 5 ms communication cycle and specifies the message transmission timing, repetition period, and priority for each ECU node, thereby establishing a solid foundation for subsequent dynamic segment scheduling and performance evaluation.

**Figure 8:**
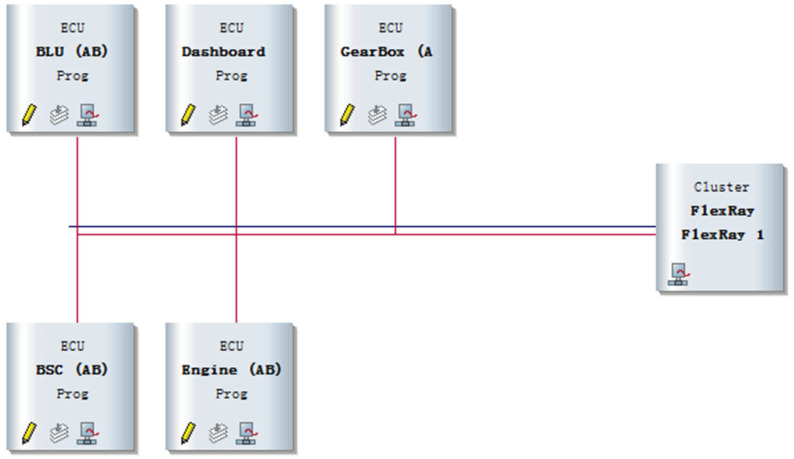
In-vehicle Network Topology Architecture.

This study utilized the CANoe.FlexRay platform to build the topology architecture of the in-vehicle network, as shown in Figure 8. In this setup, the BLU node is used to send body control information, the Dashboard node to send instrument panel information, the GearBox node to send transmission information, the BSC node to send vehicle braking information, and the Engine node to send engine operating status information. All the above nodes are connected via the FlexRay bus FlexRay 1.

**Figure 9:**
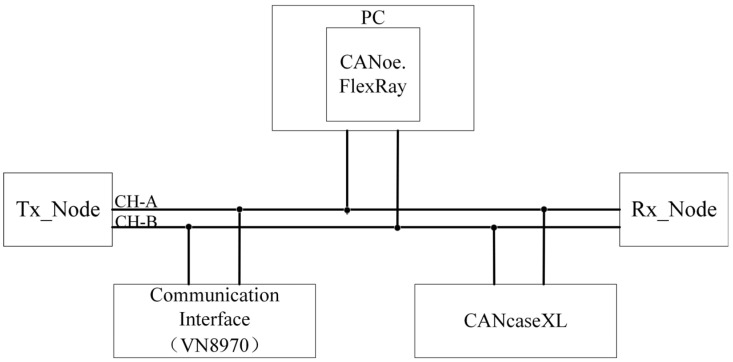
Hardware Experimental Setup Diagram.

**Figure 10:**
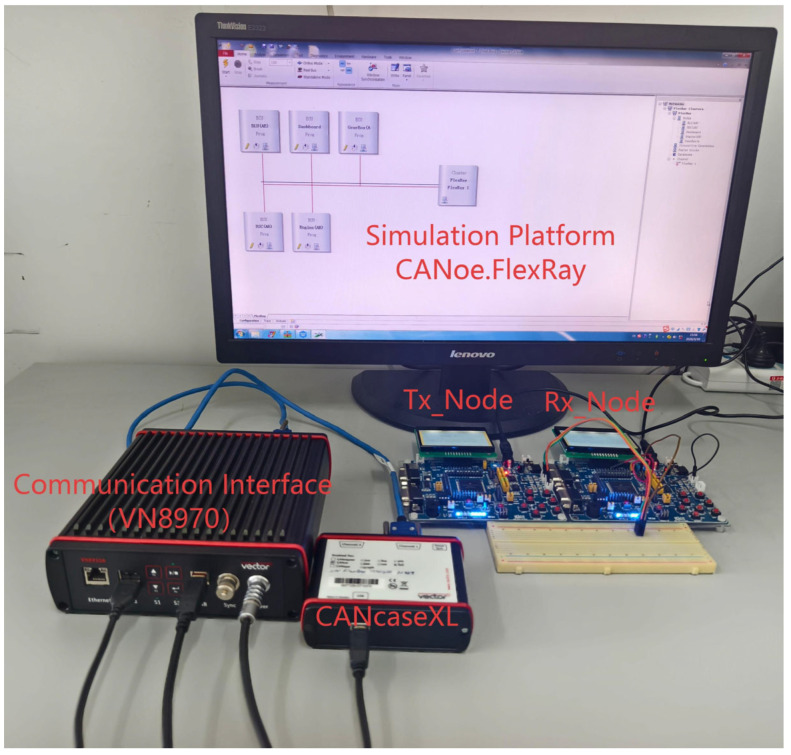
Actual Simulation Environment.

As illustrated in Figure 9, the hardware experimental setup diagram clearly presents the topology of the test platform: the PC running CANoe.FlexRay and the CANcaseXL device are both connected to the VN8970 communication interface via USB links, while the Tx Node (transmitting node) and Rx Node (receiving node) are linked to the VN8970 through twisted-pair cables, collectively forming a complete FlexRay communication hardware architecture. On this basis, this study employs CANoe.FlexRay as the core simulation platform, with CANcaseXL providing hardware authorization and real-time bus interaction support and VN8970 serving as the FlexRay communication interface. A complete in-vehicle bus simulation test environment including transmitting and receiving nodes is constructed, and the actual hardware connections and software topology are illustrated in Figure 10, which can reproduce the message transmission process of the FlexRay dynamic segment in real-vehicle scenarios.

**Figure 11:**
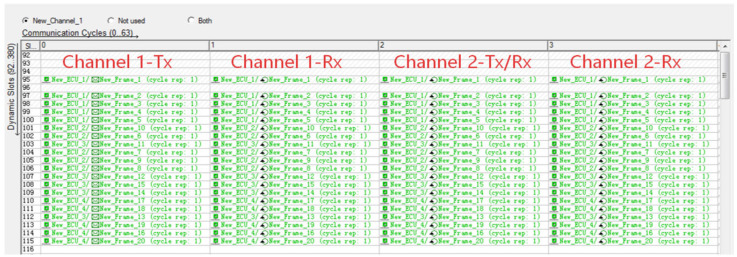
Dynamic Segment Scheduling Table.

Figure 11 illustrates the slot scheduling and message transmission mechanism of the dynamic segment in a FlexRay communication cycle. For the FlexRay bus with a dual-channel redundant architecture, the four columns in this figure correspond to the Channel 1 transmit view, Channel 1 receive view, Channel 2 redundant transmit-receive view, and Channel 2 receive view in sequence. It allocates 20 frames from 4 ECU nodes, all configured for transmission once per communication cycle. Leveraging a minislot-based event-triggered mechanism, the dynamic segment enables flexible bus bandwidth allocation and efficient delivery of non-periodic, low-priority data. Additionally, dual-channel redundant transmission and the self-monitoring behavior of transmitting nodes jointly ensure the reliability and data integrity of in-vehicle communication.

**Figure 12:**
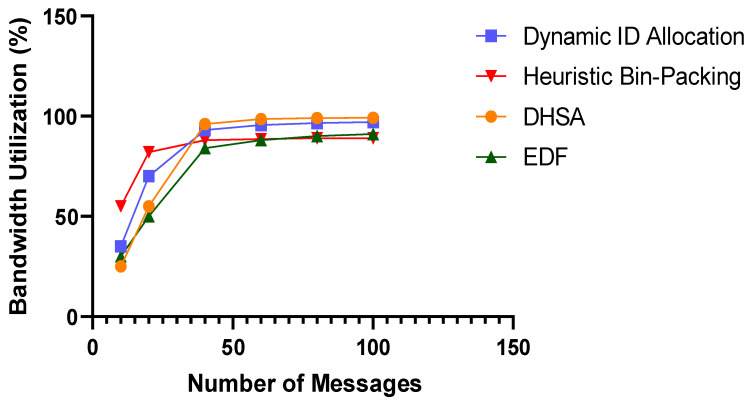
Comparison of Bandwidth Utilization for Dynamic Segment Messages.

To evaluate the performance of the DHSA proposed in this study, we conducted comparative experiments against three representative baseline algorithms: Dynamic ID Allocation, Heuristic Bin-Packing, and the classical EDF scheduling algorithm. The bus bandwidth utilization of each algorithm was measured under varying loads with different numbers of dynamic segment messages, and the results are presented in Figure 12. The experimental results show that when the number of dynamic segment messages is small, the proposed DHSA exhibits slightly lower bandwidth utilization than the Heuristic Bin-Packing algorithm while performing comparably to the EDF and Dynamic ID Allocation schemes. As the number of messages increases, however, the DHSA demonstrates a growing advantage in bandwidth utilization, achieving a utilization rate of 96.6% at the highest load tested, compared with 95.1% for Dynamic ID Allocation, 88.3% for Heuristic Bin-Packing, and only 85.16% for the EDF algorithm. These results confirm that the proposed heterogeneous scheduling algorithm for the FlexRay dynamic segment enables more efficient utilization of bus bandwidth resources and delivers superior transmission performance and resource utilization efficiency compared with the existing baseline algorithms, particularly under high-load conditions.

**Figure 13:**
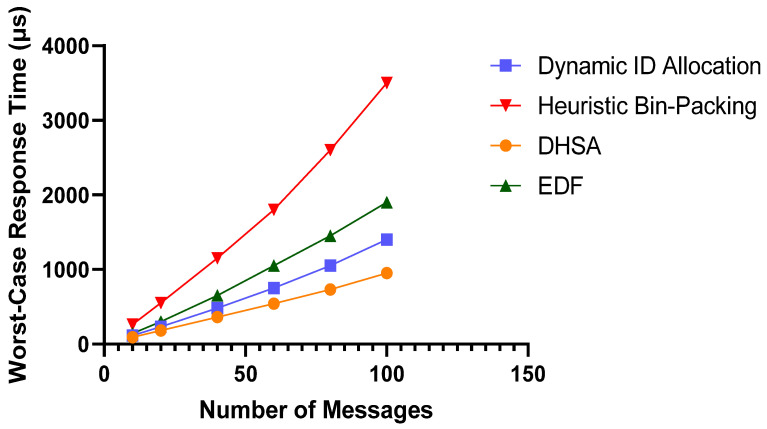
Comparison of Worst-Case Response Times for Dynamic Segment Messages.

As a core metric for evaluating real-time performance, worst-case response time directly determines the determinism and reliability of the FlexRay bus. In this experiment, we compare the worst-case response times of the proposed DHSA with three baseline schemes—Dynamic ID Allocation, Heuristic Bin-Packing, and the classical EDF algorithm—under varying numbers of dynamic segment messages, with the results shown in Figure 13. The results reveal that the worst-case response times of all algorithms increase as the number of messages grows, but the growth trend in the DHSA is the most moderate; at the peak load tested, the DHSA achieves a significantly lower worst-case response time, representing a reduction of approximately 32% compared with Dynamic ID Allocation, 50% compared with EDF, and 73% compared with Heuristic Bin-Packing, demonstrating its superior real-time performance and transmission stability under high-load conditions.

Table 1 shows the performance comparison between the proposed FlexRay dynamic segment heterogeneous message scheduling algorithm, the EDF algorithm, the Dynamic ID Allocation Algorithm, and the Heuristic Bin-Packing Algorithm. The experimental results show that the bandwidth utilization of the FlexRay dynamic segment heterogeneous message scheduling algorithm is 96.6%, whereas the Dynamic ID Allocation Algorithm’s bandwidth utilization is 95.1%, the EDF algorithm’s bandwidth utilization is 85.16%, and the Heuristic Bin-Packing Algorithm’s bandwidth utilization is 88.3%. The worst-case response time of the FlexRay dynamic segment heterogeneous message scheduling algorithm is 950 µs, while the Dynamic ID Allocation Algorithm’s worst-case response time is 1400 µs, the EDF algorithm’s worst-case response time is 1900 µs, and the Heuristic Bin-Packing Algorithm’s worst-case response time is 3500 µs.

## 5. Summary

Aiming at the message scheduling problem of the in-vehicle FlexRay bus, this paper innovatively proposes a Heterogeneous Message Scheduling Algorithm (DHSA) for the FlexRay dynamic segment. The DHSA allocates communication channels for transmitted messages through a heterogeneous allocation method of communication channels, combined with an FID allocation module to schedule messages. This improves the bandwidth utilization of message transmission while introducing a more symmetrical and balanced communication mode to the communication system. Experimental results indicate that the DHSA achieves a bandwidth utilization of 96.6%, which is higher than traditional algorithms, reducing the waste of bandwidth resources. This provides strong technical support for high-efficiency message transmission on the in-vehicle FlexRay bus in the development of intelligent connected vehicles.

## Figures and Tables

**Table 1:** Dynamic Segment Performance Comparison Table.

Performance Indicator	DHSA	Dynamic ID Allocation Algorithm	EDF Algorithm	Heuristic Bin-Packing Algorithm
Bandwidth Utilization (%)	96.6%	95.1%	85.16%	88.3%
Worst-Case Response Time (µs)	950	1400	1900	3500

## Data Availability

The data that support the findings of this study are not publicly available due to privacy concerns. Requests for access to the data should be directed to the corresponding author.
